# Identification of key immune genes of osteoporosis based on bioinformatics and machine learning

**DOI:** 10.3389/fendo.2023.1118886

**Published:** 2023-06-07

**Authors:** Song Hao, Mao Xinqi, Xu Weicheng, Yang Shiwei, Cao Lumin, Wang Xiao, Liu Dong, Hua Jun

**Affiliations:** ^1^ Department of Orthopaedics, The Second Affiliated Hospital of Soochow University, Suzhou, China; ^2^ Women’s Hospital, Zhejiang University School of Medicine, Hangzhou, China

**Keywords:** osteoporosis, bioinformatics, immunology, MSVM-RFE, diagnostic markers

## Abstract

**Introduction:**

Immunity is involved in a variety of bone metabolic processes, especially osteoporosis. The aim of this study is to explore new bone immune-related markers by bioinformatics method and evaluate their ability to predict osteoporosis.

**Methods:**

The mRNA expression profiles were obtained from GSE7158 in Gene expression Omnibus (GEO), and immune-related genes were obtained from ImmPort database (https://www.immport.org/shared/). immune genes related to bone mineral density(BMD) were screened out for differential analysis. protein-protein interaction (PPIs) networks were used to analyze the interrelationships between different immune-related genes (DIRGs). Gene Ontology (GO) and Kyoto Encyclopedia of Genes and Genomes (KEGG) analyses of DIRGs function were performed. A least absolute shrinkage and selection operation (LASSO) regression model and multiple Support Vector Machine-Recursive Feature Elimination (mSVM-RFE) model were constructed to identify the candidate genes for osteoporosis prediction The receiver operator characteristic (ROC) curves were used to validate the performances of predictive models and candidate genes in GEO database (GSE7158,GSE13850).Through the RT - qPCR verify the key genes differentially expressed in peripheral blood mononuclear cells Finally, we constructed a nomogram model for predicting osteoporosis based on five immune-related genes. CIBERSORT algorithm was used to calculate the relative proportion of 22 immune cells.

**Results:**

A total of 1158 DEGs and 66 DIRGs were identified between high-BMD and low-BMD women. These DIRGs were mainly enriched in cytokine−mediated signaling pathway, positive regulation of response to external stimulus and the cellular components of genes are mostly localized to external side of plasma membrane. And the KEGG enrichment analysis were mainly involved in Cytokine−cytokine receptor interaction, PI3K−Akt signaling pathway, Neuroactive ligand−receptor interaction,Natural killer cell mediated cytotoxicity. Then five key genes (CCR5, IAPP, IFNA4, IGHV3-73 and PTGER1) were identified and used as features to construct a predictive prognostic model for osteoporosis using the GSE7158 dataset.

**Conclusion:**

Immunity plays an important role in the development of osteoporosis.CCR5, IAPP, IFNA4, IGHV3-73 and PTGER1were play an important role in the occurrences and diagnosis of OP.

## Introduction

As a worldwide disease, osteoporosis (OP) is characterized by a decrease in bone mass and regression of bone microstructure, which lead to bone weakness and a higher risk of fracture, resulting in a great burden on society (1). In the United States, approximately 1.5 million fractures are caused by OP each year (2). It is estimated that the economic burden of OP will reach $25.3 billion by the end of 2025 in the United States alone (3). Moreover, as per the latest data in China, the estimated prevalence rates of OP were 6.46% and 29.13% in men and women over 50 years of age, respectively. With increasing age, the summary exposure value (SEV) of individuals with low bone mineral density (BMD) increased sharply from 12.78 in the 40-44 age group to a peak of 61.54 in the 85-89 age group (4). Epidemiological data on OP and fractures in various countries showed that there is a higher incidence of OP in older adults, especially postmenopausal women (5).

Physiologically, the skeletal system continuously remodels itself throughout life, and bone remodelling is completed by osteoclasts (OC), osteoblasts (OB), osteocytes and other cells. Bone remodelling begins with the resorption of mineralized bone by osteoclasts, followed by osteoblast-mediated formation of bone matrix, which is in turn mineralized (6). The osteoclastic and osteogenic processes of bone remodelling jointly maintain the stability of bone mass. However, this process is affected by age, sex, drugs, hormone levels and other factors. Once the balance of bone remodelling is broken, especially when bone resorption is stronger than bone formation, bone loss and OP will occur (7, 8).

Although OP is still considered an endocrine disease, a growing number of studies now show that it is closely related to immune and inflammatory states (9–11). Since bone and the immune system share a common developmental niche, the developmental remodelling of bone is affected by the immune system, and innate and adaptive immune cells can participate in the pathogenesis of OP by producing proinflammatory mediators (12). In bone marrow, T cells account for 5% of the total number of bone marrow cells (13). Among them, Th17 cells are mainly responsible for initiating and stimulating bone resorption (osteoclast formation) (14), while Treg cells are closely related to inhibiting bone resorption. CD8^+^ T cells inhibit the osteoclast formation process by secreting various soluble factors, such as osteoprotegerin (OPG) and interferon (IFN)-γ, which regulate bone mass (15). In addition, studies have confirmed that major histocompatibility complex class II (MHC-II) presentation imbalance is also associated with OP (11).

In this study, bioinformatics methods were used to investigate the differential gene expression profiles of OP samples in the Gene Expression Omnibus (GEO, https://www.ncbi.nlm.nih.gov/geo/) database to explore the role of inflammation in OP. Further attempts were made to identify immune-related genes as diagnostic biomarkers for OP patients, which may be helpful for the diagnosis and treatment of OP. In addition, we explored the potential relationship between immune cells and OP.

## Methods

### Data collection

**Figure 1:**
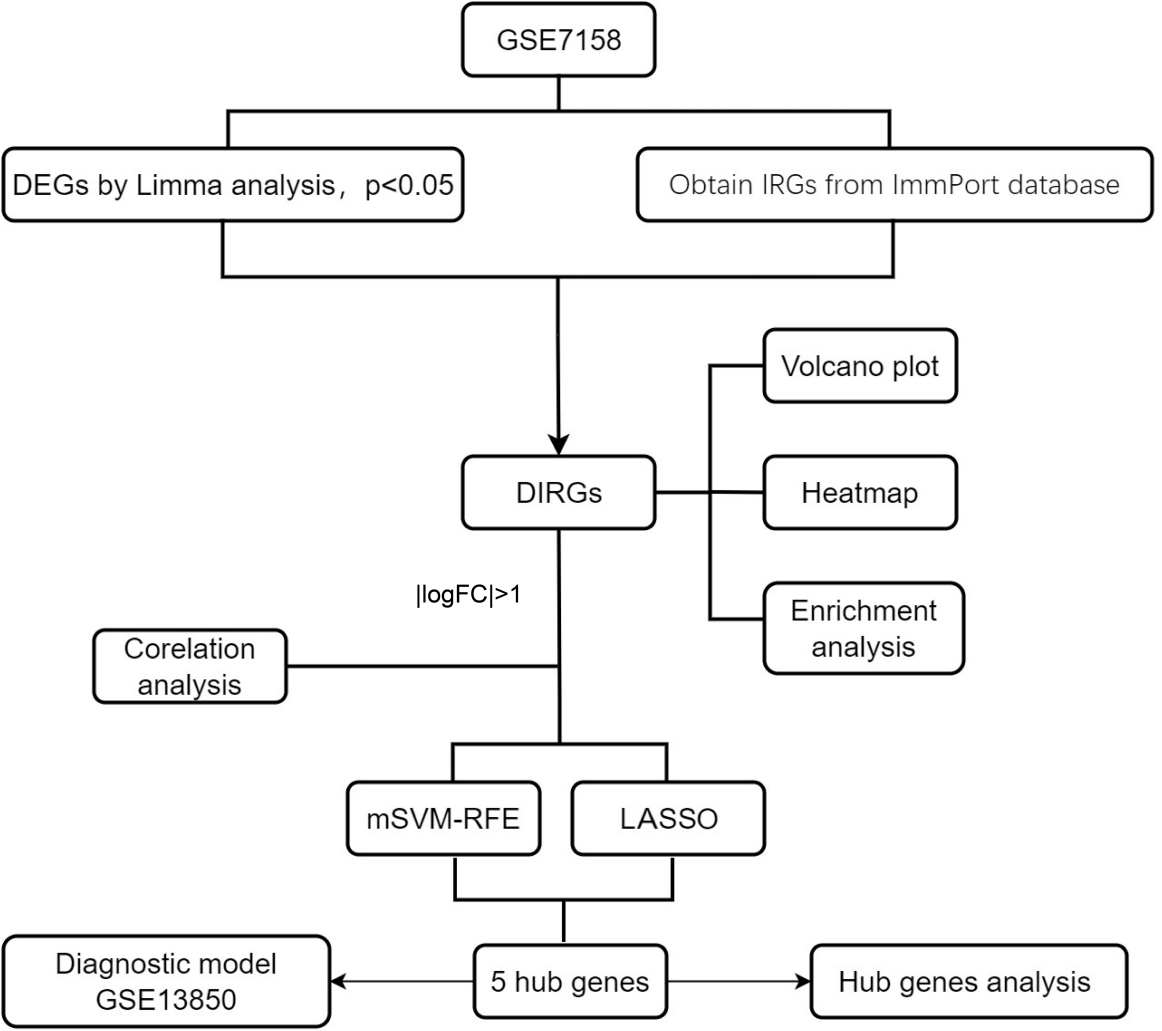
** **A flowchart showing the research methods used in this study. mSVM-RFE, multiple Support Vector Machine Recursive Feature Elimination; LASSO, Least absolute shrinkage and selection operation; logFC, log Fold Change.

The relevant OP dataset, GSE7158, was downloaded from the GEO database. This dataset, with data platform GPL570, consists of sequencing data corresponding to Homo sapiens, with 14 high-peak bone mass (HPBM) and 12 low-peak bone mass (LPBM) circulating monocyte specimen samples. Data on immune-related genes (IRGs) were downloaded from the ImmPort database (https://www.immport.org/shared/), and 1793 IRGs were obtained. The research methods used in this study are shown in **Figure 1**.

### Differential expression analysis

The “limma” R package (https://www.bioconductor.org/packages/release/bioc/html/limma.html) (16) was used to identify the differentially expressed genes (DEGs) between the HPBM and LPBM samples of the GSE7158 dataset, with the threshold of P<0.05. A total of 1158 DEGs and 1793 IRGs were further intersected to obtain differentially expressed immune-related genes (DIRGs).

### Gene ontology and Kyoto encyclopedia of genes and genomes pathway enrichment analyses

GO and KEGG pathway enrichment analyses were conducted utilizing the R package “ClusterProfiler” (17) for genes to detect the GO terms that displayed enrichment in 3 distinct categories (cellular components, molecular functions, and biological processes) and the KEGG pathways. The enrichment results were visualized by using the “ggplot2” package in R.

### Protein−protein interaction analysis

All human PPIs were obtained from the STRING (https://string-db.org/) database (18). Protein IDs were translated into gene symbols, and PPIs without corresponding gene names were eliminated. Then, the PPI network was constructed via Cytoscape 3.9.1 (https://cytoscape.org/). The cytoHubba plugin was used to identify hub genes.

### Construction of an IRG prediction model for OP

DIRGs with |log2 fold change (FC)| > 1 were selected, obtained by Spearman correlation analysis of the correlation between these genes. The least absolute shrinkage and selection operator (LASSO) and multiple support vector machine-recursive feature elimination (mSVM-RFE) algorithms were used to identify significant prognostic biomarkers in OP.

LASSO is a regression analysis algorithm used to filter variables to prevent overfitting and was performed via the “glmnet” package (19). mSVM-RFE stabilizes feature rankings by using resampling techniques at each iteration and identifies the most relevant features by deleting feature vectors generated by SVM through supervised machine learning techniques. It has a lower risk of overfitting than SVM-RFE (20). Here, the mSVM-RFE algorithm was performed using the “e1071” R package to filter the best variables.

Finally, candidate genes of the two algorithms were overlapped to obtain candidate prognostic genes of OP. Receiver operating characteristic (ROC) curves were drawn using the “pROC” R package, and the area under the ROC curve (AUC) values were calculated to evaluate the accuracy and efficiency of the candidate genes.

### Construction and validation of a nomogram model for OP diagnosis

To predict the occurrence of OP, we established a nomogram model by using the “rms” package. “Point” represents the score of the corresponding factor below, and “Total Points” represents the sum of the scores of all the above factors. Then, we plotted calibration curves to assess the predictive ability of the nomogram model.

### Quantitative real time PCR assay

Peripheral blood mononuclear cell (PBMC) were collected from DXA diagnosed osteoporosis patients and healthy controls. Total RNA was extracted by Trizol reagent (Takara), reversed by PrimeScript Reverse Transcription (RT) reagent kit (Takara), Real-time PCR was performed with an Applied Biosystems 7900HT system (Applied Biosystems) using a SYBR Premix Ex TaqTM kit (Takara). the fold change was determined using the 2−△△Ct method.

### Distribution of immune cells in OP

CIBERSORT is an analytical tool that uses gene expression data to estimate the abundance of member cell types in a mixed cell population based on linear support vector regression (SVR). As in the study by Gao et al. (21), we compared the distribution of 22 immune cells in GSE7158 between the HPBM group and the LPBM group by the CIBERSORT calculation tool.

## Result

### Expression of DIRGs in OP

**Figure 2:**
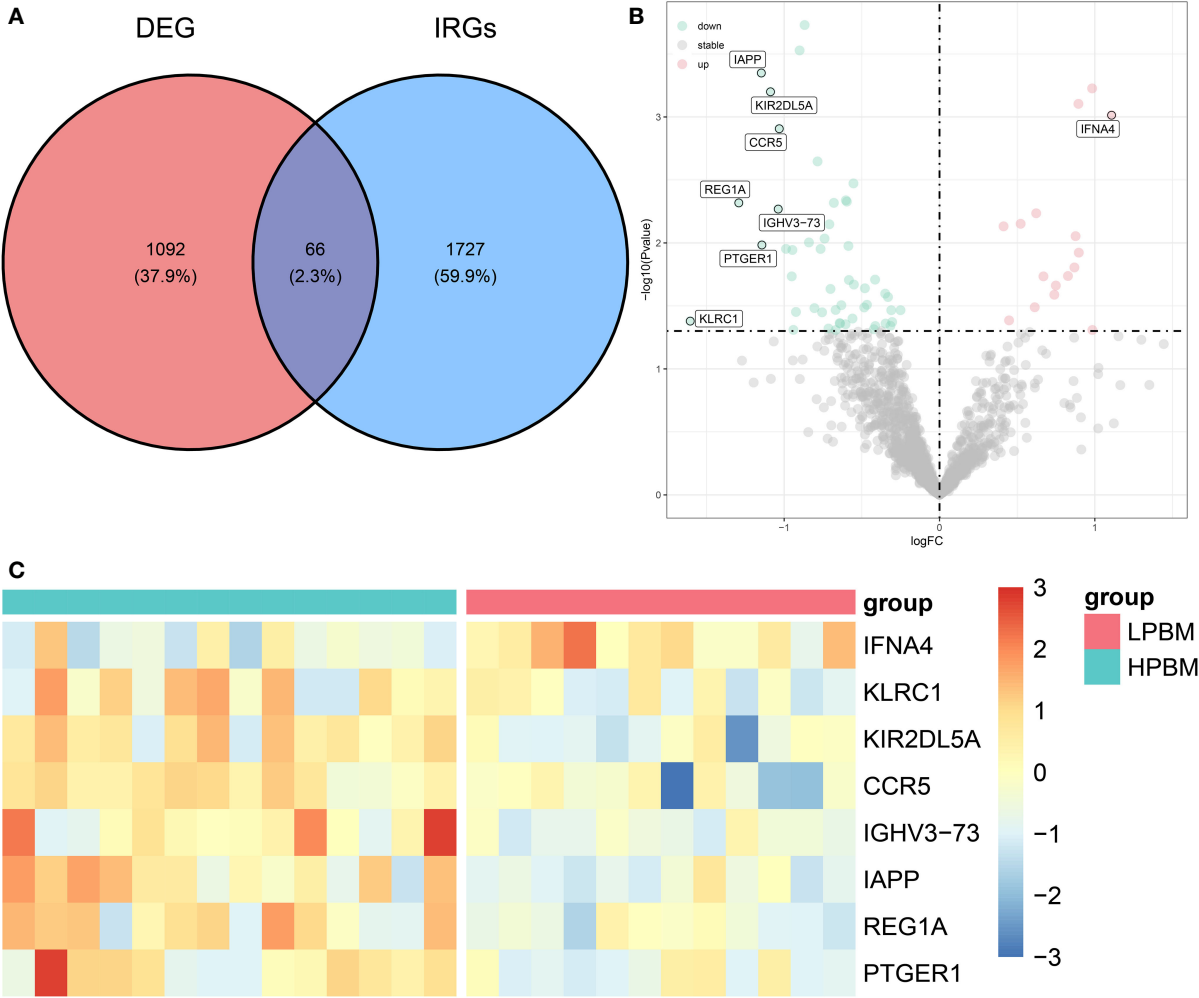
Differentially expression analysis of IRGs. **(A)** The intersection of DEGs in GSE7158 dataset and IRGs downloaded from ImmPort contains 66 DIRGs. **(B)** A volcano plot of the 66 DIRGs. **(C)** A heat map of the DIRGs identified in the GSE7158 dataset. IRGs, immune-related genes; DEGs differentially expressed genes; DIRGs, different immune-related genes.

**Figure 1** shows the flowchart associated with the analysis of DIRG expression in OP. Using the “limma” package in R, 1158 DEGs between HPBM and LPBM in the GSE7158 dataset under the criterion of p value <0.05 were obtained. We further took the intersection of 1158 DEGs and 1793 IRGs obtained from the ImmPort database to obtain 66 DIRGs (**Figure 2A**). The differences in IRG expression between HPBM and LPBM are presented as a volcano plot in **Figure 2B**, and the screened DIRGs with |log2 FC| > 1 are presented in a heatmap in **Figure 2C**. As seen from the volcano plot, among the 66 DIRGs, 16 IRGs were upregulated and 50 IRGs were downregulated in the population with low bone mass. Among the 8 IRGs shown in the heatmap, the low bone mass population had a higher IFNA4 expression level and lower IAPP expression level than the high bone mass population. In addition, KLRC1 KIR2DL5A, CCR5, IGHV3-73, REG1A, and PTGER1 had significantly lower expression in the low bone mass population.

### PPI analysis

**Figure 3:**
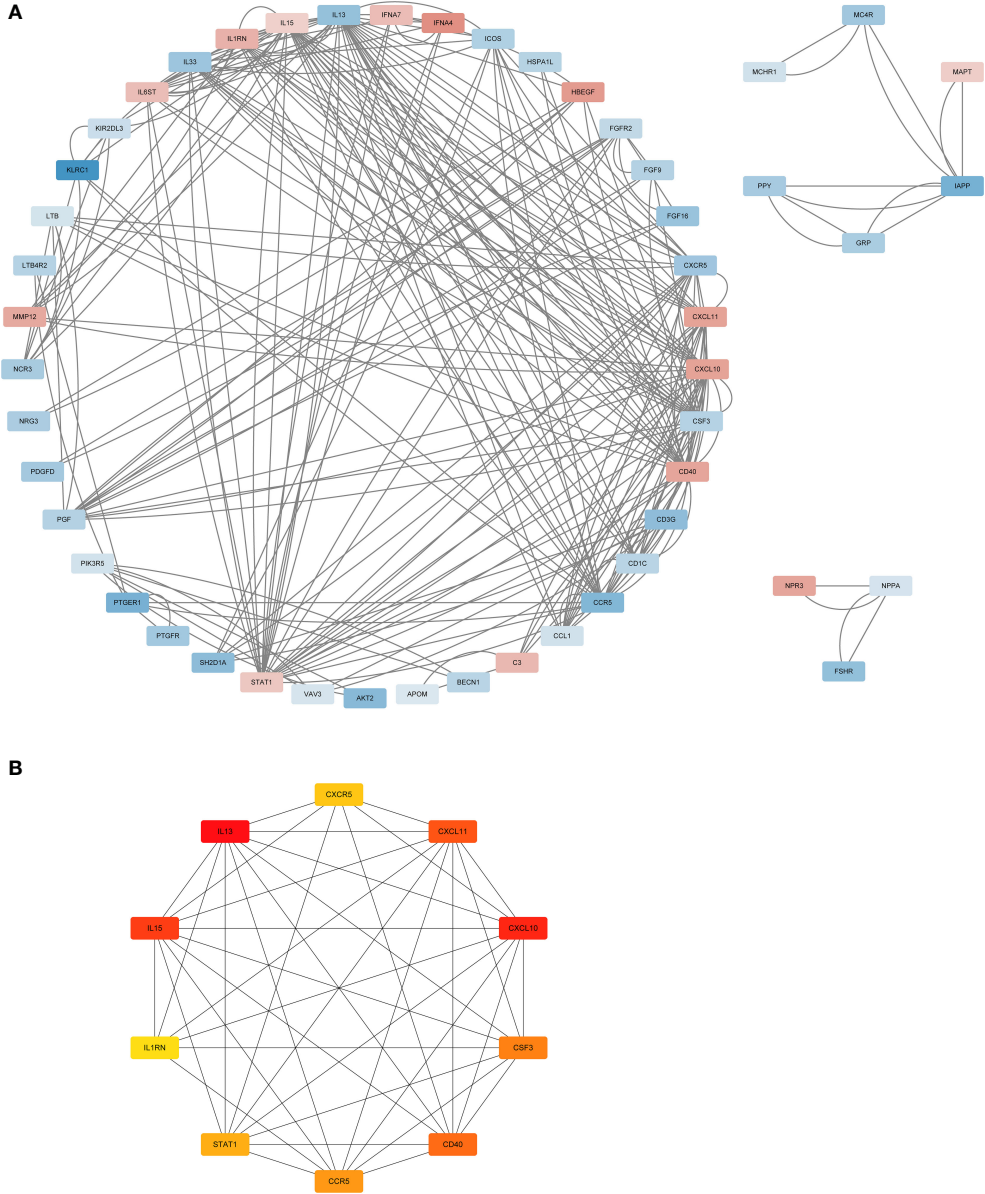
The PPI analysis. **(A)** The PPI network analysis of the 66DIRGs. **(B)** PPI network diagram of hub genes obtained by MCC algorithm. DIRGs, different immune-related genes; PPI, protein-protein interaction; MCC, Maximal Clique Centrality.

The STRING database was used to analyse the protein interactions of 66 immune genes, resulting in PPI networks with interaction scores >0.4 (**Figure 3A**). In the figure, upregulated genes are marked in red, and downregulated genes are marked in blue. Then, the cytoHubba plugin in Cytoscape software was used to cluster the network genes. The top 10 nodes in the MCC are clustered. Among them, CCR5, IL13, CXCR5 and CSF3 were downregulated, while other genes were upregulated (**Figure 3B**).

### GO and KEGG pathway enrichment analyses

**Figure 4:**
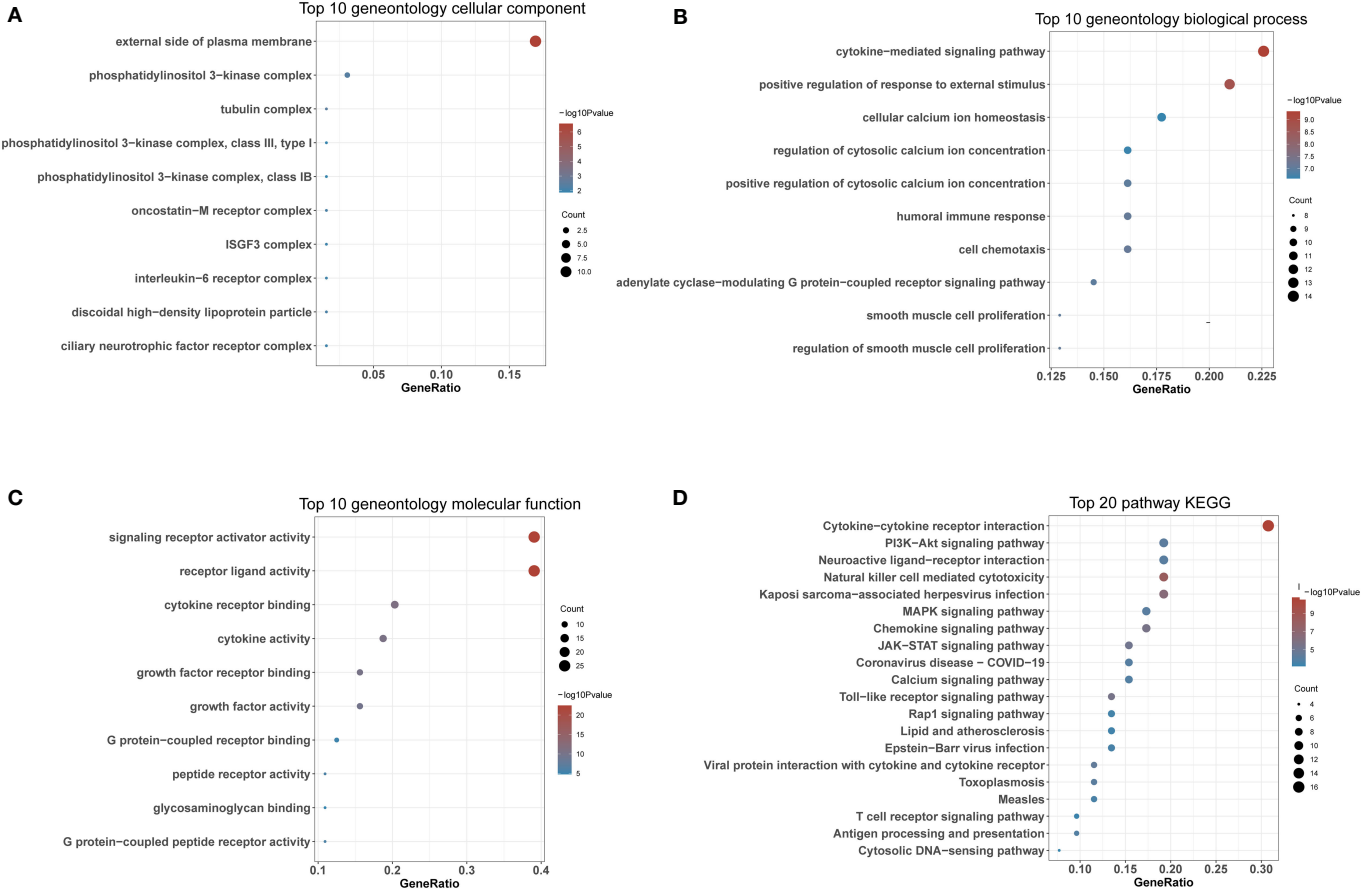
Functional enrichment of DIRGs. **(A)** CC annotation of DIRGs. **(B)** BP annotation of DIRGs. **(C)** MF annotation of DIRGs. **(D)** KEGG annotation of DIRGs, DIRGs, different immune-related genes; BPs, biological processes; CCs, cellular components; MFs; molecular functions; KEGG, Kyoto Encyclopedia of Genes and Genomes.

To further explore the enrichment pathways and functions of these 66 DIRGs, “ClusterProfiler” was used for functional enrichment analysis in R. The “ggplot2” package was used to further visualize the enrichment results. These genetic biological processes are mainly concentrated in the cytokine−mediated signalling pathway and positive regulation of the response to external stimuli, and the cellular components of the genes are mostly localized to the external side of the plasma membrane. The biological functions of the genes were mainly related to signalling receptor activator activity and receptor ligand activity (**Figures 4A–C**). The KEGG enrichment analysis indicated that the 66 DIRGs were mainly involved in cytokine−cytokine receptor interaction, the PI3K−Akt signalling pathway, neuroactive ligand−receptor interaction, and natural killer cell-mediated cytotoxicity (**Figure 4D**). These results suggest that LPBM is closely related to inflammation.

### Correlations between the expression levels of the DIRGs in OP

**Figure 5:**
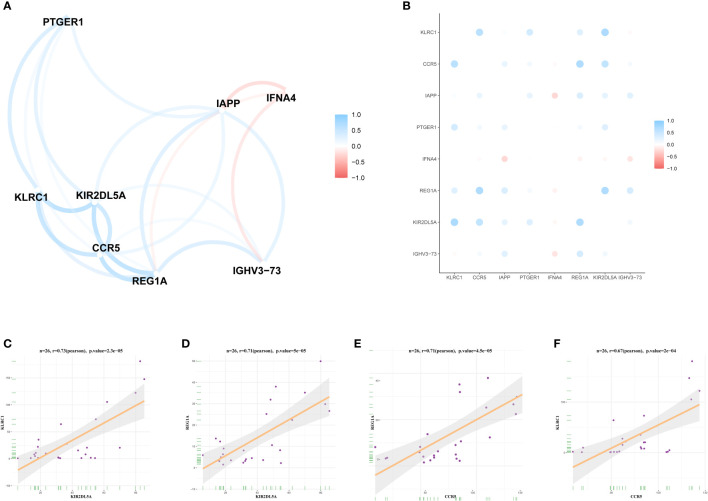
Expression correlation of DIRGs. **(A)** Heatmap of 8 DIRGs expression correlations. **(B)** DIRGs expression correlation network diagram. **(C–F)** Scatter plot of some highly correlated DIRGs. DIRGs, different immune-related genes.

To analyse the correlations between the expression levels of the DIRGs, we performed correlation analysis on the expression levels of DIRGs with |log2 FC| > 1. Here, we used the “ggcorrplot” package in R to plot the correlation analysis results as a heatmap (**Figure 5A**) and a network map (**Figure 5B**). **Figures 5C–F** shows the scatter diagrams corresponding to the four groups of genes with the strongest correlations in OP. Our results indicated that KIR2DL5A had a strong correlation with KLRC1 and REG1A, and CCR5 had a strong correlation with REG1A and KLRC1.

### Construction and assessment of the LASSO and mSVM-RFE models

**Figure 6:**
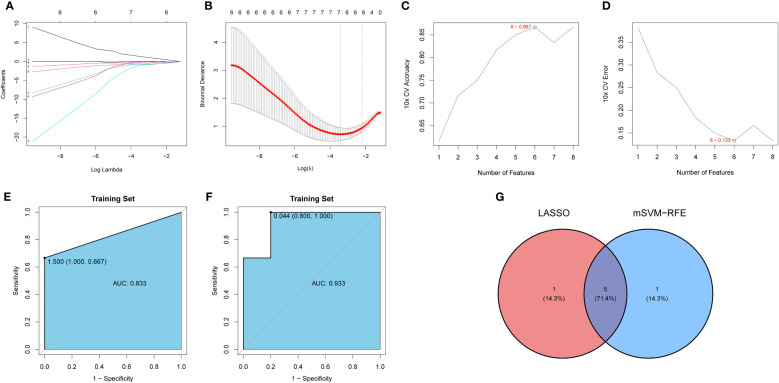
Development and verification of the model. **(A)** LASSO regression coefficient profiles of the 8 DIRGs. Each curve represents the changing trajectory of each DIRG. **(B)** Partial likelihood deviance versus log (λ)was drawn using LASSO Cox regression model. **(C)** The curve of the total within sum of squared error curve under corresponding cluster number k, and it reached the “elbow point” when k = 6. **(D)** The curve of average silhouette width under corresponding cluster number k, and the maximum of average silhouette width was achieved when k = 6. **(E, F)** ROC curves validated the performances of the LASSO regression model and the mSVM-RFE model. **(G)** Venn plots show the candidate genes by overlapping the candidate genes selected from the LASSO regression model and the mSVM-RFE model. LASSO, Least absolute shrinkage and selection operation; mSVM-RFE, multiple Support Vector Machine Recursive Feature Elimination; ROC, receiver operating characteristic; DIRG, different immune-related genes.

The LASSO and mSVM-RFE algorithms were used to identify significant prognostic biomarkers in OP. As shown in **Figures 6A, B**, the optimal λ was selected using 10-fold cross validation. Through LASSO analysis, we selected 6 DIRGs (IFNA4, KIR2DL5A, CCR5, IGHV3-73, IAPP, and PTGER1) as candidate genes from 8 DIRGs with |log2 FC| > 1. Moreover, the mSVM-RFE model was used to narrow down 8 DIRGs, and 6 DIRGs (IGHV3-73, IFNA4, CCR5, KLRC1, IAPP, and PTGER1) were selected as candidate genes (**Figures 6C, D**). ROC curves were drawn to evaluate the predictive ability of the LASSO regression and SVM-RFE models, and their respective AUC values were 0.933 and 0.833 (**Figures 6E, F**). Five DIRGs (CCR5, IAPP, IFNA4, IGHV3-73, and PTGER1) were obtained by taking the intersection of the candidate genes from the LASSO regression model and mSVM-RFE model (**Figure 6G**), which were subsequently selected as candidate genes for OP.

### Further analysis of the five important DIRGs

**Figure 7:**
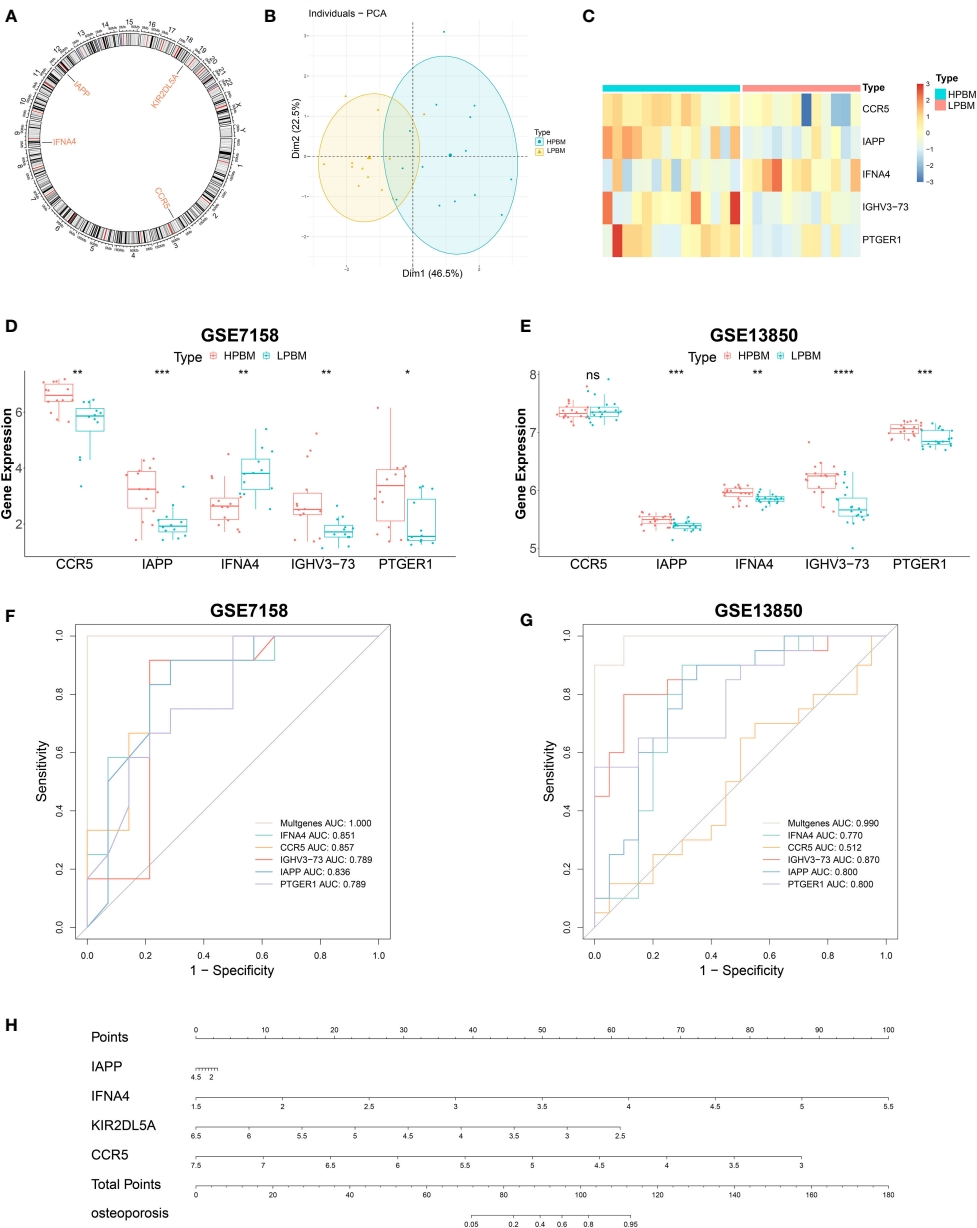
Further analysis of five important IRGs. **(A)** The chromosomal locations of five important IRGs. **(B)** Principal component analysis shows that the five genes aforementioned can clearly distinguished LPBM and HPBM. **(C)** A heat map of five important IRGs. **(D, E)** The relative expression level of five important IRGs between LPBM and HPBM from GSE7158 dataset and GSE13850. **(F, G)** ROC curves validated the performances of five important IRGs for the prediction of OP in GSE7158 and GSE13850 datasets. **(H)** diagnostic Nomo plot. IRGs, immune-related genes; LPBM, low-peak bone mass; HPBM, high-peak bone mass; ROC, receiver operating characteristic. (*P<0.05, **P<0.01, ***P<0.001, ****P<0.0001). ns, no significance.

Five candidate genes (CCR5, IAPP, IFNA4, IGHV3-73, and PTGER1) obtained from the intersection of the LASSO regression model and mSVM-RFE model were selected for further analysis. **Figure 7A** shows the chromosomal positions of CCR5, IAPP, IFNA4, IGHV3-73 and PTGER1. As shown in the results of principal component analysis (**Figure 7B**), the five candidate genes could clearly distinguish LPBM from HPBM, indicating that they may play a key role in the diagnosis of OP. In addition, the predictive ability of the five candidate genes was further tested in the training group (GSE7158) and the validation group (GSE13850). As shown in **Figures 7C, D**, in the GSE7158 dataset, compared with that in HPBM women, the expression of CCR5, IAPP, IGHV3-73, and PTGER1 was downregulated, while the expression of IFNA4 was upregulated in LPBM women. In the GSE13850 dataset, the expression of IAPP, IGHV3-73, and PTGER1 was higher in high-BMD women than in low-BMD women, whereas the results observed for IFNA4 were opposite to those observed in the GSE7158 dataset (**Figure 7E**). Furthermore, the AUC value of the ROC curve of the five DIRGs (GSE7158, multigene, AUC = 1.000; GSE13850, multigene, AUC = 0.990) showed that they had a stronger ability to predict OP than a single gene (**Figures 7F, G**). Subsequently, we constructed an OP risk-related nomogram (**Figure 7H**), which can be used as a function of the risk score to distinguish between healthy and OP samples.

### RT-qPCR validation

**Figure 8:**
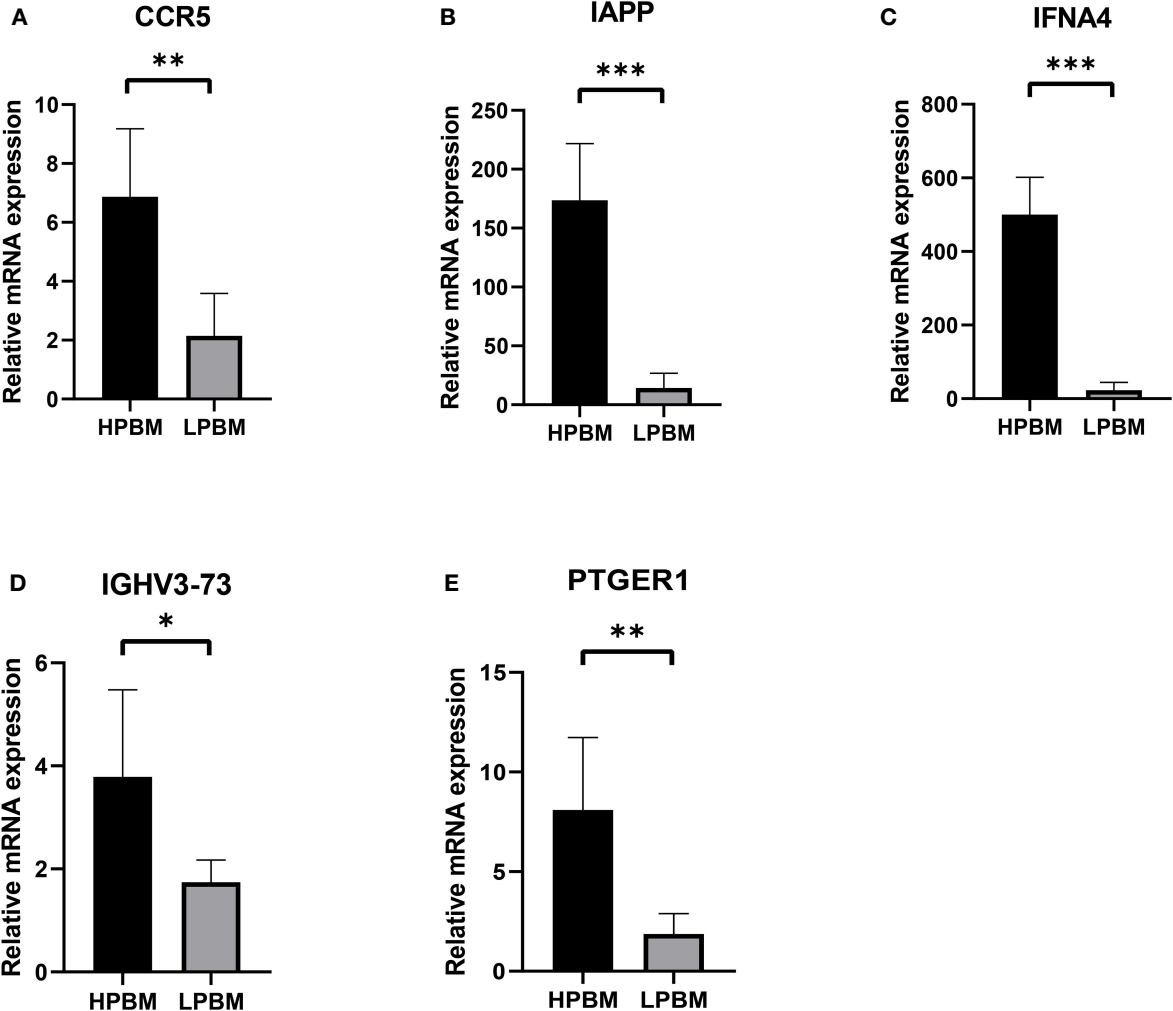
The expression of CCR5, IAPP, IFNA4, IGHV3-73, PTGER1 **(A-E)** in the HPBM and LPBM group. LPBM, low-peak bone mass; HPBM, high-peak bone mass. (*P<0.05, **P<0.01, ***P<0.001).

In order to verify hub genes *in vivo*, PBMC mRNA isolated from whole blood was used to detect the expression of five central genes (CCR5, IAPP, IFNA4, IGHV3-73, and PTGER1) identified by our bioinformatics analysis. A total of RT-qPCR results were collected and showed that, The mRNA expression levels of five central genes were lower in the osteoporosis group than in the healthy control group (**Figure 8**). The expression levels of CCR5, IAPP, IFNA4, IGHV3-73, and PTGER1 were consistent with the results of bioinformatics analysis.

### Distribution of immune cells

**Figure 9:**
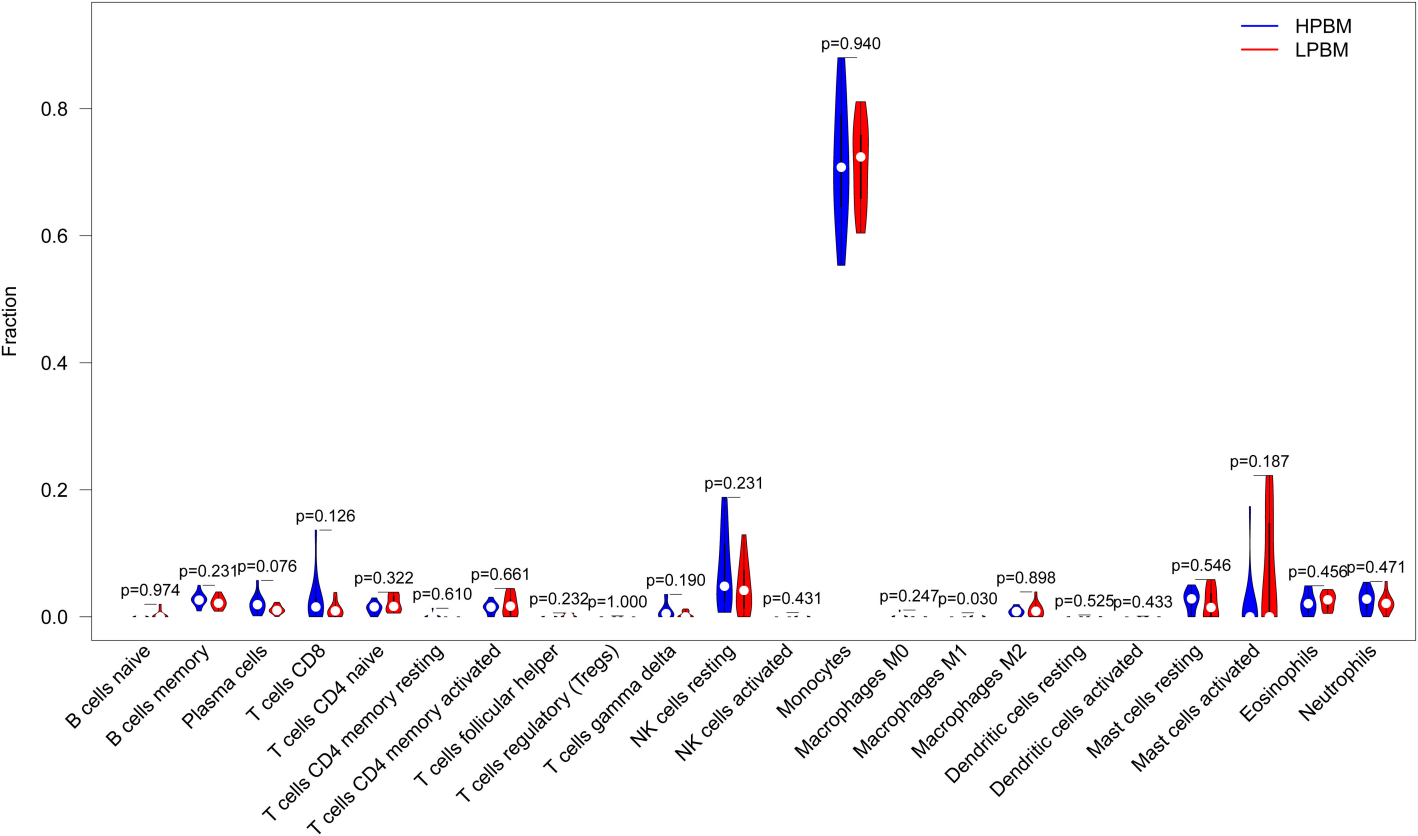
Differences of the infiltrate immune cells between the LPBM and HPBM group. LPBM, low-peak bone mass; HPBM, high-peak bone mass.

To better understand the relationship between inflammation and OP, we calculated the relative proportions of 22 immune cell types in each sample. Then, we compared the infiltration of 22 immune cells between LPBM and HPBM samples. We found that the infiltration abundance of M1 macrophages in LPBM samples was significantly higher than that in HPBM samples, while the infiltration abundance of other immune cells was not significantly different (**Figure 9**).

## Discussion

Osteoporosis is the result of an imbalance in the highly balanced physiological process of “bone remodeling”.Classical theory hold that osteoporosis is essentially a bone remodeling disorder caused by estrogen deficiency, aging, low body weight, secondary diseases, drugs (7).However, with the in-depth understanding of the relationship between osteoporosis and the immune system, it is gradually discovered that the immune system is intricately involved in bone physiology and pathology. As early as 1997, W B Ershler et al. found that the immune system can participate in the regulation of osteoclasts through pro-inflammatory cytokines (such as IL-1 and IL-11) (22), With decades of development, in 2018 Srivastava et al. proposed the field of “Immunoporosis”,to emphasize the role of immunity in osteoporosis (23).

It is well known that bone structure is composed of collagen, matrix proteins, calcium hydroxyapatite crystals and cellular components. The cellular components of bone include osteoblasts (OBs), osteoclasts (OCs), osteocytes (OYs), stromal cells, mesenchymal stem cells (MSCs), hematopoietic stem cells (HSCs), etc. Osteoblasts and osteoclasts play a major role in maintaining bone homeostasis. OBs, derived from MSCs, can produce type-I collagen and matrix proteins to promote the deposition of calcium hydroxyapatite. OCs are mainly derived from monocytes and promote bone demineralization through hydrolytic enzymes, thus inhibiting the osteogenic proces (24). Any factor that disrupts bone balance may lead to abnormal bone metabolism. In osteoporosis, osteoclast activity increases and osteogenic activity is relatively insufficient, resulting in net bone loss.

The immune system is involved in osteoporosis in a number of ways. Macrophages, monocytes, and dendritic cells can function by acting as precursors to osteoclasts (25). Macrophages exhibit different phenotypes in different tissue microenvironments. The M1 phenotype is a classic inflammatory activated macrophage that acts as an osteoclast precursor. The M2 phenotype is selectively activated macrophages.It is involved in osteogenic metabolism by stimulating bone marrow mesenchymal stem cells to differentiate into OBs. The M1 and M2 phenotypes are usually associated with bone catabolism and anabolism, respectively (10, 11). One study showed that M1/M2 macrophage ratio increased in OVX osteoporosis mouse model (26), reflecting that estrogen deficiency mediated macrophage ratio changes may be closely related to osteoporosis.

In addition, other immune cells are also involved in osteoporosis, such as NK cells and lymphocytes (ILCs), mainly as producers of pro-inflammatory mediators involved in the development of osteoporosis (27, 28). Neutrophils can raise promoting osteoporosis cell chemotaxis (such as Th17) promotes the development of osteoporosis (29).

Inflammatory mediators such as reactive oxygen species (ROS), chemokines and cytokines can directly or indirectly participate in the process of bone metabolism through NF-kB and PI3k/AKT pathways, classic WNT/beta-catenin pathways, STATs and SMAD pathways. (30, 31).

In order to further explore the relationship of osteoporosis and immune genes,This paper comprehensively analysed the expression and regulatory mechanism of IRGs in patients with different bone masses and established a diagnostic model of IRGs in OP patients through machine learning.

GO/KEGG enrichment analysis indicated that PI3K/AKT signalling pathway is involved in osteoporosis. As previous studies showed that the PI3K/AKT signalling pathway promotes OB generation and osteogenesis (32). In one experiment, mice with double knockout of Akt1 and Akt2 showed delayed ossification (33). Takashi et al. found that Runx2 enhanced PI3K-AKT signalling by increasing the protein levels of the PI3K subunit and Akt, and enhanced PI3K-AKT signalling promoted Runx2 binding to Runx2-dependent transcribed DNA (34). In addition, the Akt/FOXO3a signalling pathway can affect the inflammatory signalling pathway to regulate OB proliferation (35). This evidence suggests that IRGs can affect bone mass changes by regulating the PI3K-AKT signalling pathway. The deterioration of bone structure is closely related to the immune processes mediated by cytokines. Proinflammatory cytokines such as interleukin-6 (IL-6), tumour necrosis factor-α (TNF-α) and interleukin-1 (IL-1) can accelerate bone loss by promoting bone resorption. Cytokines affect the osteogenic process through direct or indirect processes (9).

Some of these stimulate the secretion of other cytokines, acute phase proteins, proteases, and noncytokines. The effects of cytokines on osteoclasts are usually mediated by the M-CSF and RANKL/RANK/OPG signalling pathways. Inhibition of osteoblast proliferation, maturation and activity was achieved by inhibition of the RUNX2 and WNT/β-catenin signalling pathways (36).

NK cell monitoring systems include various activation and inhibition receptors on the cell surface, which can help identify and kill target cells through antibody-dependent cell-mediated cytotoxicity (ADCC) effects (37). NK cells can be activated by IL-15, thereby killing OCs (38) and playing a role in the regulation of OP.

Five genes (CCR5, IAPP, IFNA4, IGHV3-73 and PTGER1) were screened by the LASSO regression and mSVM-RFE models based on eight IRGs with the largest differences between the two bone mass groups. CCR5 is a receptor for chemokine CCL5, which is expressed in T cells, smooth muscle endothelial cells, epithelial cells and even parenchymal cells (39). It is associated with HIV infection, cell proliferation, migration, angiogenesis, metastasis and survival (40). CCR5 is specifically expressed in periosteal skeletal stem cells (P-SSCs). Treatment with CCL5 induces P-SSC migration *in vivo* and bone healing, while CCL5/CCR5 deletion, CCR5 inhibition, or local P-SSC ablation reduces the number of osteoblasts and delays bone healing (41). Our study found decreased CCR5 expression in the low bone mass group, suggesting that OP may be related to CCR5-mediated chemotactic function. Islet amyloid polypeptide (IAPP or amylin) is a hormone secreted by islet β-cells that is prone to deposition to form an insoluble amyloid precipitate (42). IAPP is involved in diabetes, Alzheimer’s disease and cardiovascular diseases (43). The IAPP receptor is a heterodimer of the calcitonin receptor and RAMP. In an IAPP-knockout animal model, bone mass was decreased, and osteoclasts were increased (44). Dacquin et al. found that bone resorption was increased in mice with IAPP deficiency, but the osteogenesis process was not significantly affected (45). In this study, the expression level of IAPP in patients with reduced bone mass was lower than that in patients with high bone mass, which was similar to the findings of Dacquin et al. However, the reason why IAPP caused decreased bone mass and increased osteoclasts needs to be further studied. IFNA4 is a type I interferon (IFNαβ) that is mainly involved in antiviral immunity. It is rapidly produced after viral infection and induces the production of other IFN-αisoforms in fibroblasts (46). In addition, IFNA may be involved in autoimmune diseases such as systemic lupus erythematosus (47). Our study found that the increase in IFNA4 was significantly different in the experimental group with low bone mass, but the opposite conclusion was obtained in the validation group. Thus, the effect of IFNA4 on bone homeostasis needs to be further studied. IGHV3-73 is a subfamily of the immunoglobulin heavy chain gene (IGHV), which is involved in the V region of the variable domain of the immunoglobulin heavy chain for antigen recognition (48). IGHV is often used in the diagnosis of chronic lymphocytic leukaemia (49). Our study found that IGHV3-73 expression was downregulated in LPBM. PTGER1 is one of the four prostaglandin receptors involved in biological processes such as immunity, inflammation and pain conduction (50). In addition, PTGER1 plays a unique role in the regulation of bone homeostasis. Loss of PTGER1 leads to inactivation of Hif1α and an increase in the oxygen consumption rate, thereby increasing osteoblast differentiation (51). Zhang et al. found that EP1 (-/-) mesenchymal progenitor cells isolated from bone marrow have a high ability to differentiate into osteoblasts and accelerate the formation and mineralization of bone nodules *in vitro* (52). Our study found that the expression of PTGER1 in the LPBM group was lower than that in the HPBM group, which was different from the role of PTGER1 knockdown in promoting osteogenesis. This may be because although pge2 stimulation at low concentrations for a short time can promote the osteogenesis process through the PTGER1 receptor pathway, it may change the expression ratio of the pge2 receptor in osteoblasts and the responsiveness of osteoblasts to pge2 in the long-term inflammatory state, resulting in the shift of bone homeostasis to bone resorption, resulting in bone mass reduction. To verify the expression of these five genes in healthy patients and patients with osteoporosis, we collected PBMC and confirmed that these five genes were closely related to the development of osteoporosis by RT-qPCR.

Then, we established a diagnostic model of OP using the above five genes and drew the ROC curve to verify its performance in the test dataset. The model was found to have high diagnostic value, so we constructed a nomogram model based on these five genes for OP risk prediction. In clinical work, blood samples of hospitalized patients are easy to obtain for disease diagnosis and the judgement of disease changes. This nomogram has clinical value for OP risk screening.

Finally, using the CIBERSORT algorithm, we found that compared with the HPBM group, M1 macrophages were mainly distributed in the LPBM group, and the results were significantly different, while there was no significant difference in the distribution of other immune cells. M1 macrophages can mediate the inflammatory state by producing a variety of proinflammatory cytokines (53), which is consistent with our experimental results.

There are also some shortcomings in our study. The expression levels of CCR5, IAPP, IFNA4, IGHV3-73 and PTGER1 have been verified by RT-qPCR, and flow cytometry is still needed to further detect the mechanism of action of these molecules. Due to the limited sample size, the nomogram model may need further testing before clinical application.

## Conclusion

In conclusion, we investigated the potential correlation between immune inflammation and the occurrence of OP through machine learning and found a strong relationship between the two. Some immune-related genes such as CCR5, IAPP, IFNA4, IGHV3-73 and PTGER1 were lowly expressed in LPBM.The proportion of M1 macrophages in the LPBM group was higher than that in the HPBM group.

## Data availability statement

Publicly available datasets were analyzed in this study. This data can be found here: Data generated or analyzed during this study are available in the following repositories: [GEO : GSE7158, https://www.ncbi.nlm.nih.gov/geo/query/acc.cgi?acc=GSE7158]; [GEO : GSE13850, https://www.ncbi.nlm.nih.gov/geo/query/acc.cgi?acc=GSE13850]; [ImmPort, https://s3.immport.org/release/genelists/GeneList.txt?download=true].

## Ethics statement

The studies involving human participants were reviewed and approved by The Ethics Committee of The Second Affiliated Hospital of Soochow University. The patients/participants provided their written informed consent to participate in this study.

## Author contributions

SH: Conceptualization, Methodology, Software. MX: Writing Original draft preparation. XW: Data curation. WX: Visualization, Investigation. HJ: Supervision. YS: Software, Validation. LD, CL: Writing Reviewing and Editing. Sincere thanks to HJ, LD and WX for their help of the work. SH and MX contributed equally to this study and shared first authorship. All authors contributed to the article and approved the submitted version.
